# Gambling Among Finnish 14–16-Year-Old Adolescents Before (2008–2009), During (2010–2011), and After (2013–2017) Setting the Legal Age Limit of 18 for Gambling and the Role of Socio-Economic Status

**DOI:** 10.1007/s10899-021-10091-6

**Published:** 2021-12-07

**Authors:** Tiina Latvala, Tomi Lintonen, Pauliina Luopa, Susanna Raisamo

**Affiliations:** 1 Finnish Institute for Health and Welfare, Helsinki, Finland; 2 The Finnish Foundation for Alcohol Studies, Helsinki, Finland

**Keywords:** Gambling, Legislation, Trend, Socio-economic status, Inequality

## Abstract

Legislation prohibiting minors from engaging in gambling is a gambling policy measure set to protect adolescents from the harmful effects of gambling. The Finnish gambling system is based on a state monopoly, regulated by the Lotteries Act. After an amendment to the Lotteries Act, the new minimum legal gambling age was raised to 18 years old between 2010 and 2011. The main purpose of this study was to discover how the amendment to the act altered adolescents’ gambling (14–16-year-olds) and to examine whether the amendment decreased socio-economic differences. Adolescents gambling was studied before (2008–2009), during (2010–2011), and after (2013–2017) the age limit of gambling was raised in Finland. The study based on five waves (2008–2009, 2010–2011, 2013, 2015, 2017) of the national repeated cross-sectional School Health Promotion Study. Cross-tabulations where gambling was studied by study year and socio-economic status (SES) were formulated, and the statistical differences were studied by using χ^2^-tests. Percentage change in gambling frequency was also examined by study year and SES. Study years were analyzed separately to model the weekly gambling via logistic regression models. Adolescent gambling significantly decreased over time. It appears that raising the legal gambling age had a permanent effect on under-aged gambling. However, differences in gambling by adolescents’ family’s SES increased during the study period, indicating widening inequalities in gambling among adolescents. Diminishing inequalities in adolescent gambling is likely to require both societal action and consensus on adolescent gambling being a significant social and public health concern.

## Introduction

Internationally in most jurisdictions there is legislation prohibiting minors from engaging in gambling. Age limits are set to protect adolescents from the harmful effects of gambling and to secure the wellbeing and favorable development of adolescents. The legislation prohibiting underage children and adolescents from gambling is a gambling policy measure and, as such, a concrete example of a social norm dictating that gambling should not be a part of the lives of children and adolescents. However, youth are increasingly exposed to gambling from a broad range of media (Monaghan et al., 2008). By growing availability and commercialization, gambling is widely visible and socially acceptable in Western countries nowadays (Monaghan et al., 2008; Smith et al., 2020). Especially adolescents, who are familiar with using the Internet and other digital media, are in a vulnerable position (Clemens et al., 2017; King et al., 2010). Despite the age limit of 18 being common in many jurisdictions, gambling has been shown to be prevalent among adolescents under 18 years old (Blinn-Pike et al., 2010; Volberg et al., 2010). Particularly among boys and adolescents with a higher weekly income, gambling seems to be more common (Blinn-Pike et al., 2010; Buja et al., 2019; Darling et al., 2006).

Gambling is common in Finland and, when compared with other countries, what is exceptional in Finland is the wide supply of slot machines in convenient locations, such as supermarkets, kiosks, gas stations, and cafés (Matilainen, 2017). Slot machine gambling is also the most popular type of gambling among Finnish youth (Raisamo et al., 2015). The Finnish gambling system is based on a state monopoly, regulated by the Lotteries Act. After an amendment to the Lotteries Act, the new minimum legal gambling age was raised from 15 years old to 18 years old in October 2010. However, slot machines were given a transition time in which to change the age limit and the new age limit for slot machines took effect on July 1, 2011. After the change in legislation, the six-month prevalence of slot machine gambling among 12–16-year-olds decreased from 44% in 2011 to 13% in 2013 (Raisamo et al., 2015). Also, overall gambling declined among Finnish minors between 2011 and 2017 (Raisamo et al., 2020), and there is also some evidence of a decrease in problem gambling (Nordmyr & Österman, 2016). Besides raising the age limit, the aim of the amendment was also to improve the supervision of age limits and forbid gambling advertisements that are directed at adolescents. Nevertheless, the supervision of the age limit of gambling has been shown to be weak in Finland (Warpenius et al., 2016).

One of the most important goals of health policy should be to reduce inequalities among people by diminishing gap between socio-economic classes. Adolescents’ socio-economic status (SES) can be conceptualized in many ways, for example, by combining the parental educational level, occupational status, and income. A low level of parental education and reduced access to material resources can have a negative influence on adolescents’ health-related quality of life (Von Rueden et al., 2006) and SES has been linked with mental health outcomes and general health symptoms (Quon & McGrath, 2014).

Among adults, low SES is associated with problem gambling (van der Maas, 2016), and a similar result has been seen among adolescents (Fröberg et al., 2015; Tozzi et al., 2013; Welte et al., 2008). It has also been shown that, together with impulsivity, SES increases the risk of gambling onset among youths (Auger et al., 2010). However, greater family wealth has been linked to more symptoms of disordered gambling (Elgar et al., 2018). While problem gambling is associated with low SES, studies have only found weak associations (McComb & Sabiston, 2010) or found non-existing associations (Cheung, 2014) between non-problem gambling and SES (measured by family income and/or parental education). Also, community-level inequalities exist in adolescent gambling as at-risk/problem gambling has been shown to be higher for adolescents living in more disadvantaged regions (Gori et al., 2014) and in regions with the highest income inequality (Canale et al., 2017).

Besides being a social problem, adolescent gambling must be considered as significant public health concern (Messerlian et al., 2005). Adolescent gambling, especially gambling that occurs on weekly basis, is associated with problem gambling and risk behaviors, such as substance use, poorer health, and even violent acts (Räsänen et al., 2015a, 2015b, 2015c, 2017). Even adolescent who gambled less often than once a month smoked, used snuff and alcohol more frequently than adolescent who had not gambled during previous year. Also bullying, delinquent acts and drunkenness related drinking was more common among them (Räsänen et al., 2015b). Further, higher gambling frequency was positively associated with signs of school burnout, staying up late, and having more than 1 sexual partner (Räsänen et al., 2015a).

The main purpose of this study is to discover how the amendment to the act altered adolescents’ gambling, whether there are differences in gambling according to the adolescents’ socio-economic background, and to examine whether the amendment decreased socio-economic differences. Adolescents gambling was studied before (2008–2009), during (2010–2011), and after (2013–2017) the age limit of gambling was raised in Finland. From a public health perspective, it is important to study the trends of socioeconomic differences in adolescents’ gambling as it can deepen our understanding of the socioeconomic inequalities of adolescent gambling and enable us to identify target populations when planning and implementing interventions.

## Methods

### The Participants and Procedure

The study was based on the national repeated cross-sectional School Health Promotion Study. Data were based on five waves (2008–2009, 2010–2011, 2013, 2015, 2017). Changes in gambling among adolescents was studied before (2008–2009), during (2010–2011), and after (2013–2017) raising the age limit of gambling in Finland. The School Health Promotion Study of the Finnish Institute for Health and Welfare is a school-based survey designed to examine the health, health behaviors, and school experiences among eighth (14–15 years old) and ninth graders (15–16 years old) in Finland (Matikka et al., 2015). The survey was conducted biennially in different parts of Finland and the pooled two-year data (2008–2009 and 2010–2011) represent the whole country. After 2011, the survey was conducted every second year over the whole country.

In the School Health Promotion Study, the data is collected by a questionnaire that is completed anonymously during a school lesson under the supervision of a teacher. The questionnaire takes 30 to 45 min to complete and was executed between 2008 and 2013 by paper and pencil and after that by computer, on the Internet. In the Internet questionnaire, participants used usernames and passwords that could not be identified with personal data. In both cases it was made sure that answering was confidential, and no one could not see the participants’ answers. The participants were informed about the nature of the study as well as the voluntary nature of participation. The School Health Promotion Study has been accepted by the ethics committee of Pirkanmaa Hospital District (2008–2011) and the Finnish Institute for Health and Welfare (2013–2017).

The 2008–2009 study covered 82% of Finnish eighth and ninth grade students, the 2010–2011 study covered 80% of them, the 2013 study covered 84%, the 2015 study covered 43%, the 2017 study covered 64%, and the 2019 study covered 75%. The exact number of participants in each wave is shown in Table 1.

**Table 1:** Distribution of gambling frequency, parental education and employment status of family by study year

	Boys (N = 262,693)					Girls (N = 262,221)				
Year	2008–2009^a^	2010–2011	2013^a^	2015^a^	2017^a^	2008–2009^a^	2010–2011	2013^a^	2015^a^	2017^a^
Participants (N)	54,433	51,329	50,223	25,147	37,152	54,216	51,216	49,255	25,257	37,392
Gambling frequency										
Gambling at least once a week	38.7***	36.3	11.4***	13.2***	13.1***	5.2	5.0	1.2***	1.3***	1.4***
Gambling less often than once a week	42.9***	45.2	36.4***	31.1***	30.0***	35.4***	38.0	12.4***	12.2***	6.9***
No gambling	18.4	18.4	52.2***	55.6***	56.9***	59.5***	57.0	86.5***	86.5***	91.7***
Parental education										
Low	4.7***	7.0	6.9	5.2***	4.6***	4.8***	8.2	6.7***	4.4***	4.1***
Medium	25.7***	29.7	27.3***	25.5***	25.0***	30.6***	28.5	26.6***	25.1***	24.9***
High	69.6***	63.3	65.7***	69.3***	70.5***	64.6***	63.3	66.7***	70.5***	71.0***
Unemployment in the family										
Yes	25.5***	30.1	28.1***	31.6***	29.6	27.0***	31.8	29.7***	33.6***	32.7**
No	74.5	69.9	71.9	68.4	70.4	73.0	68.2	70.3	66.4	67.3

^a^Statistical difference is calculated between study year and year 2010–2011 ***p ≤ 0.001, **p ≤ 0.01

### Measures

In survey gambling was defined as following: Gambling is playing, where prize or loss is money. Gambling games are for example betting, slot machines, scratch cards, gambling games in Internet (internet poker) and private card games with money stake. Gambling was enquired about using the question “How often do you gamble?” In the 2008–2009 study, the five response alternatives were “daily or almost daily,” “1–3 times a week,” “approximately 2–3 times a month,” “once a month or less often,” and “I have not gambled during the previous year.” After the year 2009, the response alternatives were changed to “6–7 days per week,” “3–5 days per week,” “1–2 days per week,” “less than once a week,” “less than once a month,” and “I have not gambled during the previous year.” These alternatives were combined into “once a week,” “less than once a week,” and “I have not gambled during the previous year.”

The socioeconomic variables that were investigated were parental education level and unemployment in the family over the past 12 months. Parental education level was categorized as *low* (only basic education), *medium* (vocational school-level education), or *high* (college-level/academic education), based on the parent with the higher level of education. Unemployment in the family over the past 12 months was dichotomized as “none” or “one or both parents.”

### Statistical Analysis

The distributions of gambling frequency and socioeconomic variables among boys and girls from 2008 to 2017 were studied using cross-tabulation, and the statistical differences of gambling frequencies between study years were studied by using χ^2^-tests (see Table 1). The percentage change in gambling frequency was studied by using this formula: (a-b)/b*100%), where *a* stands for the percentage of gambling in the study year and *b* for the percentage of gambling during 2008–2009. Cross-tabulations where gambling was studied by study year and SES were formulated, and the statistical differences of gambling frequencies among SES classes between study years were studied by using χ^2^-tests (see Table 2). Percentage change in gambling frequency was also examined by study year and SES (see Table 3). Study years were analyzed separately to model the weekly gambling via logistic regression models. The results of the logistic regression models are presented as odds ratios (ORs) and their 95% confidence intervals (CIs) (see Table 4). Models in which the grade level of adolescents were adjusted were performed separately for boys and girls in order to explain weekly gambling by SES. The “employed with a high level of education” category was used as a reference category. Analyses were done using IBM SPSS Statistics for Windows version 27.0.

**Table 2:** The proportion (%) of gambling frequency among adolescents by study year and SES background

Gambling frequency	Boys							Girls						
	SES1	SES2	SES3	SES4	SES5	SES6	χ^2^-test, df = 5	SES1	SES2	SES3	SES4	SES5	SES6	χ^2^-test, df = 5
2008–2009														
Weekly	37.6	40.4	39.1	40.9	38.2	50.4	84.6***	3.8	5.6	5.5	7.3	6.6	13.4	292.3***
Less often	44.2	40.9	43.3	43.3	40.9	31.3	83.4***	33.6	36.6	36.5	41.7	36.4	35.2	137.5***
No gambling	18.2	18.7	17.6	15.8	20.9	18.3	25.8***	62.5	57.7	57.9	51.0	57.0	51.4	305.8***
2010–2011														
Weekly	34.7	38.4	35.4	39.3	36.6	47.0	133.4***	3.6	5.4	4.8	6.7	7.2	11.9	334.0***
Less often	46.6	43.9	47.0	43.9	41.5	37.2	84.7***	36.7	39.6	38.5	42.2	36.6	39.8	67.5***
No gambling	18.7	17.7	17.5	16.8	21.9	15.9	37.7***	59.8	55.0	56.7	51.1	56.2	48.4	210.1***
2013–2017														
Weekly	10.6	11.8	10.8	11.9	21.4	34.4	1553.0***	0.8	1.1	1.0	1.2	3.7	8.2	1359.3***
Less often	35.3	33.3	32.4	31.2	26.3	23.6	285.4***	10.2	10.9	10.3	11.5	10.6	11.4	20.0***
No gambling	54.0	54.9	56.8	56.9	52.9	42.1	222.2***	89.0	88.1	88.8	87.4	85.7	80.3	214.5***

*SES1*, High education employed; *SES2*, High education unemployed; *SES3*, Medium education employed; *SES4*, Medium education unemployed; *SES5*, Low education employed; *SES6*, Low education unemployed

**Table 3:** Percentage change in gambling between 2008–2009 and study period by SES bakground

Percentage change in gambling	2010–2011 versus 2008–2009	2013 versus 2008–2009	2015 versus 2008–2009	2017 versus 2008–2009	2010–2011 versus 2008–2009	2013 versus 2008–2009	2015 versus 2008–2009	2017 versus 2008–2009
High education employed								
Weekly	− 7.7	− 75.3	− 69.1	− 68.6	− 5.3	− 81.6	− 78.9	− 78.9
Less than weekly	+ 5.4	− 12.7	− 23.3	− 28.1	+ 9.2	− 64.6	− 64.0	− 80.4
No gambling	+ 2.7	+ 186.3	+ 199.5	+ 209.9	− 4.3	+ 39.8	+ 39.4	+ 48.2
High education unemployed								
Weekly	− 5.0	− 72.0	− 69.6	− 70.3	− 3.6	− 82.1	− 80.4	− 80.4
Less than weekly	− 7.3	− 11.2	− 24.0	− 23.7	− 8.2	− 66.4	− 64.5	− 79.0
No gambling	− 5.3	+ 180.2	+ 202.1	+ 202.1	− 4.7	− 50.3	− 49.0	− 58.1
Medium education employed								
Weekly	− 9.5	− 74.2	− 70.6	− 70.8	− 12.7	− 85.5	− 80.0	− 80.0
Less than weekly	− 8.5	− 17.3	28.2	− 30.7	+ 5.5	− 66.0	− 69.6	− 82.5
No gambling	− 0.6	+ 207,4	+ 242.0	+ 233.0	− 2.1	+ 49.9	+ 51.6	+ 59.9
Medium education unemployed								
Weekly	− 3.9	− 71.9	− 69.4	− 70.7	− 8.2	− 84.9	− 86.3	− 80.8
Less than weekly	+ 1.4	− 19.6	− 31.6	− 37.4	+ 1.2	− 67.6	− 68.6	− 82.0
No gambling	+ 6.3	+ 240.5	+ 266.5	+ 284.8	+ 0.2	+ 67.5	+ 68.4	+ 78.6
Low education employed								
Weekly	− 4.2	− 49.7	− 31.7	− 39.8	+ 9.1	− 47.0	− 21.2	− 51.5
Less than weekly	+ 1.5	− 28.9	− 39.6	− 49.1	+ 0.5	− 67.3	− 67.0	− 81.9
No gambling	+ 4.8	+ 147.4	+ 135.4	+ 168.9	56.2	84.5	83.5	90.2
Low education unemployed								
Weekly	− 6.7	− 39.9	− 16.5	− 29.4	− 11.2	− 50.0	− 18.7	− 30.6
Less than weekly	+ 18.8	− 9.3	− 41.5	− 41.5	+ 13.1	− 60.8	− 68.5	− 81.0
No gambling	− 13.1	+ 125.7	+ 116.4	+ 151.9	− 5.8	+ 54.7	+ 51.8	+ 63.4

**Table 4:** Weekly gambling (ORs with 95% CIs) by study year, gender and SES background

Gambling at least once a week	Boys					Girls				
	2008–2009	2010–2011	2013	2015	2017	2008–2009	2010–2011	2013	2015	2017
High education employed	1	1	1	1	1	1	1	1	1	1
High education unemployed	**1.1 (1.1–1.2)**	**1.2 (1.1–1.2)**	**1.2 (1.1–1.3)**	1.1 (1.0–1.2)	1.0 (0.9–1.1)	**1.5 (1.3–1.7)**	**1.5 (1.4–1.7)**	**1.5 (1.2–2.0)**	1.3 (0.9–1.8)	1.3 (1.0–1.7)
Medium education employed	1.1 (1.0–1.1)	1.0 (1.0–1.1)	1.1 (1.0–1.2)	1.0 (0.9–1.1)	1.0 (0.9–1.1)	**1.5 (1.3–1.6)**	**1.4 (1.2–1.5)**	1.2 (0.9–1.6)	1.3 (0.9–1.9)	1.3 (0.9–1.8)
Medium education unemployed	**1.1 (1.1–1.2)**	**1.2 (1.1–1.3)**	**1.3 (1.1–1.4)**	1.1 (0.9–1.2)	1.0 (0.9–1.2)	**2.0 (1.7–2.2)**	**1.9 (1.7–2.2)**	**1.6 (1.2–2.3)**	1.2 (0.8–1.9)	**1.7 (1.2–2.4)**
Low education employed	1.0 (0.9–1.1)	1.1 (1.0–1.2)	**2.3 (2.1–2.7)**	**2.7 (2.2–3.3)**	**2.3 (1.9–2.7)**	**1.8 (1.4–2.2)**	**2.1 (1.7–2.5)**	**5.3 (3.9–7.3)**	**6.8 (4.3–10.5)**	**3.9 (2.5–6.3)**
Low education unemployed	**1.7 (1.5–1.9)**	**1.7 (1.5–1.9)**	**4.3 (3.7–4.8)**	**5.6 (4.7–6.7)**	**4.2 (3.5–4.9)**	**3.9 (3.2–4.7)**	**3.6 (3.1–4.2)**	**10.5 (8.0–13.7)**	**15.1 (10.8–21.2)**	**12.4 (9.1–16.9)**

*p* values ≤ 0.05 are given in bold

## Results

### The Participants

Among the adolescents, the proportion of males was 50.1% and the percentage of eighth graders was 49.9%. At the time of the surveys, the eighth graders were 14–15 years old and the ninth graders were 15–16 years old. About a third of the adolescents lived in a family where one or both parents were unemployed or laid off, and more than half lived in a family where one or both parents had a high level of education (see Table 1).

### Changes in Gambling From 2008 to 2017

After the study period 2010–2011, weekly gambling decreased and non-gambling increased for every year (see Table 1: 2010–2011 vs. 2013: χ^2^ = 21,120, *df* = 2, *p* ≤ 0.001; 2010–2011 vs. 2015: χ^2^ = 15,062, *df* = 2, *p* ≤ 0.001; 2010–2011 vs. 2017: χ^2^ = 22,371, *df* = 2, *p* ≤ 0.001). For example, 36% of boys gambled on a weekly basis in 2010–2011 but in 2017 the percentage was only 13% (χ^2^ = 5418, *df* = 1, *p* ≤ 0.001). The decrease in weekly gambling was most pronounced between the years 2008–2009, and the year 2013 for both sexes. Among boys, the percentage change was 71 and among girls, 77. From the year 2013 to 2017, weekly gambling significantly increased from 11 to 13% among boys (χ^2^ = 55.2, *p* ≤ 0.001). Overall, boys gambled more often than girls in every year (2008–2009: χ^2^ = 25,702, *df* = 2, *p* ≤ 0.001; 2010–2011: χ^2^ = 22,317, *df* = 2, *p* ≤ 0.001; 2013: χ^2^ = 13,802, *df* = 2, *p* ≤ 0.001; 2015: χ^2^ = 6118, *df* = 2, *p* ≤ 0.001; 2017: χ^2^ = 10,952, *df* = 2, *p* ≤ 0.001) (see Table 1).

**Fig. 1:**
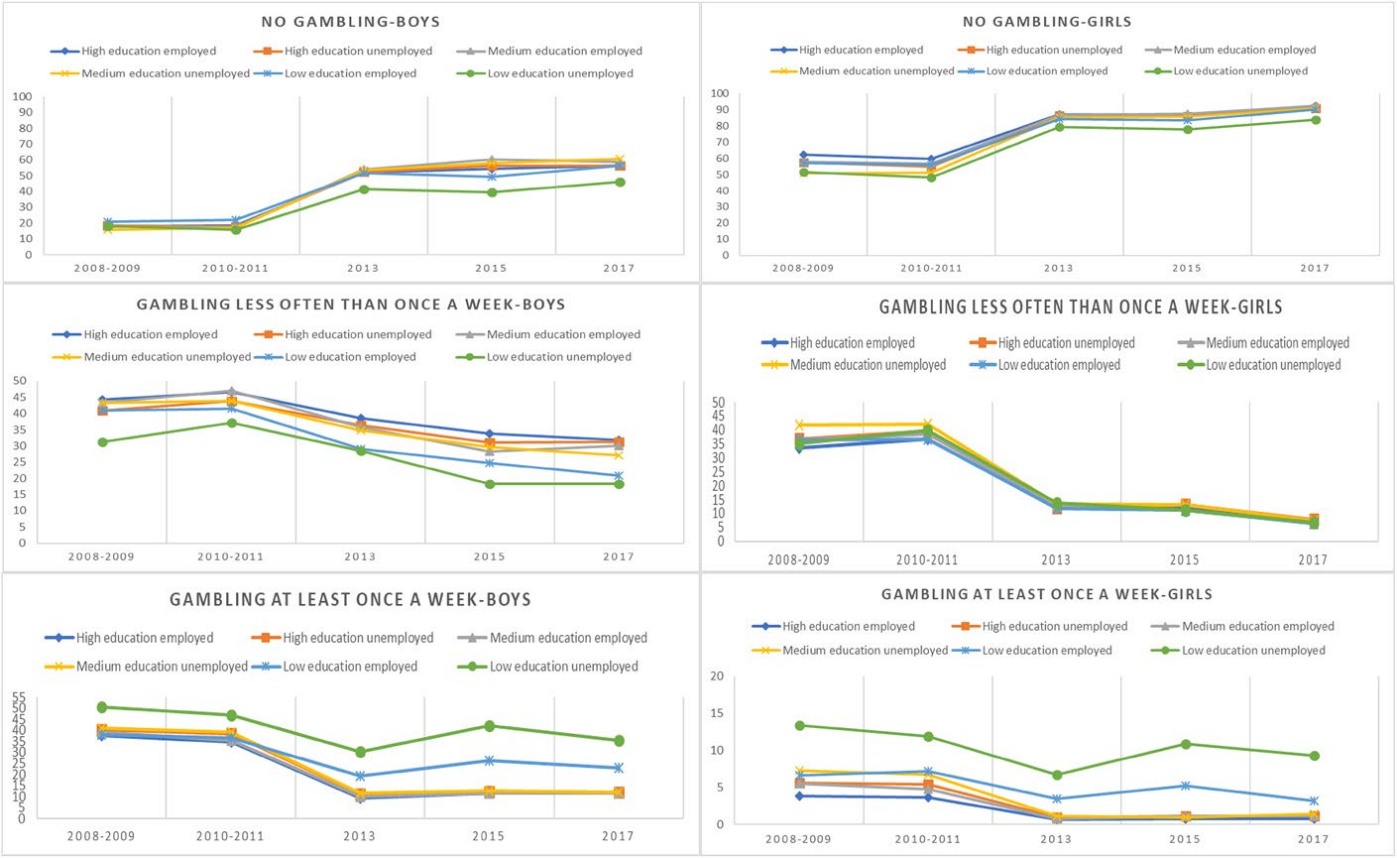
Gambling frequency based on adolescent socio-economic background

When gambling was examined by SES indicators (parents’ education and employment status), gambling decreased, and non-gambling increased after 2010–2011 in every year among all SES classes (see Table 2, Fig. 1). However, the decrease in the percentage change was lower among adolescents who had parents with a low level of education compared to those with highly educated parents (see Table 3). The percentage change in weekly gambling during 2008–2009 versus 2017 was 69% among boys with highly educated, employed parents and 29% among boys with unemployed parents with a low level of education. For girls with highly educated, employed parents, the percentage change was 79% and among girls with unemployed parents with a low level of education, it was 31% (see Table 3). Overall, in every year, weekly gambling was more common and the gambling frequency of less than once a week was lower among adolescents who had unemployed parents with a low level of education compared with other SES classes (see Table 2). Since the year 2013, weekly gambling was also more common among adolescents with employed parents with a low level of education (see Tables 2, 3, Fig. 1).

When the risk of weekly gambling was examined by sex, SES, and study year, it was found that, among adolescents with parents with a low level of education, the risk of weekly gambling increased after the period 2010–2011 among boys and girls (see Table 4). Among girls with unemployed parents with a low level of education, the risk of weekly gambling was 12 times as high compared with girls with highly educated, employed parents in the year 2017 (see Table 4).

## Discussion

This study showed that the adolescent gambling of 14–16-year-olds in Finland significantly decreased over time (2008–2017). It appears that raising the minimum legal gambling age from 15 to 18 years old had a permanent and pronounced effect on under-aged gambling. However, differences in gambling by adolescents’ family’s SES increased during the study period, indicating widening inequalities in gambling among adolescents. The decrease in the percentage change in gambling was lower among adolescents who had parents with a low level of education compared with adolescents whose parents were employed and had a medium or high level of education. Overall, weekly gambling was more common before, during, and after amending the legislation among adolescents who had unemployed parents with a low level of education. After the change in the legislation, adolescents with employed parents with a low level of education also gambled more often on a weekly basis. Also, the risk of weekly gambling increased among adolescents with parents with a low level of education. Differences in gambling in Finland are also seen among adolescents with different educational tracks. The School Health Promotion Study has shown that gambling is more common among adolescents in vocational schools than among those in eighth and ninth grades and upper secondary schools. Increasing inequality in gambling among socio-economically disadvantaged youth is very concerning as weekly gambling is also linked with problem gambling, poorer health, and risk taking (Räsänen et al., 2015a, 2015b, 2017). Co-occurring risk behaviors can be harmful because of their negative influence on an adolescent’s growth, life course, and overall health.

Higher SES can have divergent influences on adolescent gambling. On the one hand, the higher disposable income that is related to a higher SES might enable adolescents to gamble more (Buja et al., 2019; Darling et al., 2006). However, in this study, infrequent gambling (i.e., gambling less than once a week) was more common among adolescents with a higher SES. On the other hand, parents who are more educated might see gambling as a serious concern related to their adolescents’ health and therefore be more eager to supervise their adolescents’ gambling than those who are less privileged. It has been shown that low levels of parental monitoring are associated with the risk of getting involved in gambling and developing gambling problems (Spångberg & Svensson, 2020; Vachon et al., 2004). Further, parental approval of gambling is also linked with increased adolescent gambling (Leeman et al., 2014). Additionally, parents’ engagement in gambling may vary based on SES. Weekly gambling expenditure was highest among Finnish adults who had vocational school as their highest level of education and unemployed people spent more on gambling than employed people (Salonen et al., 2017). There is also extensive empirical support for associations between parental gambling and adolescent gambling (McComb & Sabiston, 2010). Unfortunately, our data did not allow us to explore parental attitudes on gambling or parental gambling.

Research on inequalities in adolescent gambling is still limited. Studies have found associations between problem gambling and low SES (Fröberg et al., 2015; Tozzi et al., 2013; Welte et al., 2008), and some studies have found a weak, or even non-existing, relationship between low SES and non-problem gambling (Cheung, 2014; McComb & Sabiston, 2010). Apart from being due to the different measurements of SES, this inconsistency might be due to the cultural differences of the study populations and differences in gambling legislation and policy. Gambling legislation and policy can differently influence the gambling participation of adolescents coming from diverse SES groups. Change in inequality is also dependent on time because the social context varies. Thus, the examination of trends in relationships between SES and gambling provides important information on the socioeconomic inequalities of adolescent gambling. Yet, as far as we known, this was the first study that examined changes in adolescent gambling and SES over time.

It is important to study changes in adolescent gambling as there are still a very limited number of studies on the time trends of adolescent gambling (Raisamo et al., 2020). This would require surveys to be conducted regularly among adolescents. In Finland there are no gambling surveys that are targeted solely towards adolescents. Adolescent gambling is studied by a survey that is conducted among the whole Finnish population and the number of adolescents in this survey is relatively small. There are also three surveys—the School Health Promotion Study (Matikka et al., 2015), the European School Survey Project on Alcohol and Other Drugs (ESPAD, 2020), and the Adolescent Health and Lifestyle Survey (Räsänen et al., 2015c)—designed to examine both the health and health behaviors of adolescents, but these contain only one question, or no more than a few questions, regarding adolescent gambling.

One of the values of this study is the large sample size that allowed us to study gambling participation between both sexes and SES classes. However, in 2015, the survey covered only 43% of Finnish adolescents, which was significantly less than in other study years. This study may slightly underestimate gambling frequency among youth because the survey was conducted among adolescents attending school. Those students who were truant or absent were excluded and they may be more likely to gamble than students attending school. Also, as mentioned earlier, this survey was not intended to measure adolescents gambling behavior; it included only one question on gambling frequency. Further, the response alternatives for the question concerning gambling were changed after 2009, which enabled us to create more precise gambling frequency classes. Further, the data was based on self-reported measures, so we cannot rule out the under- or overestimation of gambling. Also, our measure of SES was not based on any objective indicator and was a subjective measure that reflected different aspects of socioeconomic position.

Information on gambling trends among adolescents is important for health policy. The increase in non-gambling, as well as the decrease in gambling, that occurred once a week or less among Finnish adolescents after raising the age limit was in line with other Finnish studies that used a different dataset (Raisamo et al., 2015, 2020). However, this trend is also seen in other Western countries, even when there were no changes in the legal age of gambling (Gambling Commission, 2016, 2018; Stinchfield, 2011; Volberg et al., 2010). Thus, the decrease cannot be explained solely by legislation, but it most definitely had a significant role in it. There might be several explanations for why the prevalence of adolescent gambling has decreased, including changes in income, marketing, prevention, and in adults’ gambling. It might be that the novelty of commercial gambling has worn off and youth are more interested in other activities, like networking on the Internet and video game playing (Stinchfield, 2011). However, this question needs to be addressed in future research. In Finland raising the gambling age limit decreased adolescent gambling, but socio-economic differences increased. After the amendment, weekly gambling was more common, and the risk of weekly gambling increased among adolescents who had parents with a low level of education. Also, the percentage change in weekly gambling was lower among these adolescents. Used alone, age limits for gambling are not an effective way to stop minors from gambling, especially if the supervision of the age limit for gambling is weak (see Warpenius et al., 2016). It would be important to initiate prevention programs in which these socio-economic differences are noted. Overall, more information should be given to the possible harms associated with adolescent gambling, especially to parents and to those working with adolescents. Diminishing inequalities in adolescent gambling is likely to require both societal action and consensus on adolescent gambling being a significant social and public health concern.
